# Awareness, Acceptability, and Perceived Effectiveness of Text-Based Therapy Among Graduate Students: Cross-sectional Study

**DOI:** 10.2196/34102

**Published:** 2022-07-07

**Authors:** Samari A Blair, Andrea N Brockmann, Kelsey M Arroyo, Chelsea A Carpenter, Kathryn M Ross

**Affiliations:** 1 College of Medicine University of Florida Gainesville, FL United States; 2 Social and Behavioral Sciences Program College of Public Health and Health Professions University of Florida Gainesville, FL United States; 3 Department of Clinical and Health Psychology College of Public Health and Health Professions University of Florida Gainesville, FL United States

**Keywords:** mental health, text-based therapy, graduate students

## Abstract

**Background:**

Research has suggested that there is a mental health crisis occurring among graduate students in the United States. Moreover, many students go without effective treatment owing to the limited availability of mental and behavioral health resources on college campuses. Text-based therapy may represent a viable method for increasing access to mental health support for graduate students, but little is known regarding its acceptability in this population.

**Objective:**

The purpose of this study was to assess how graduate students perceive text-based therapy and their likelihood of seeking out this form of therapy.

**Methods:**

In total, 265 graduate students completed a cross-sectional web-based survey that included multiple-choice and open-ended questions assessing their perceptions of text-based therapy and the likelihood of seeking out this form of therapy. Chi-square tests, ANOVAs, and nonparametric Wilcoxon signed-rank tests were used to examine differences in multiple-choice questions. The constant comparative method was used for qualitative analyses of the open-ended question responses.

**Results:**

Participants (n=265) were predominately non-Hispanic White (166/265, 62.6%) and female (167/265, 63%) with a mean age of 28.3 (SD 5.1) years. Over half of the participants (139/265, 52.5%) were not aware that text-based therapy existed; however, 65.3% (173/265) reported that they would consider using text-based services, if available. In comparison to face-to-face therapy, participants reported being less likely to seek out text-based therapy and perceived it as less effective (*P*<.001). Qualitative results indicated that participants were concerned about the ability to effectively communicate and build rapport through text-based therapy and thought that this modality may be more effective for some mental and behavioral health concerns than others. Moreover, participants noted that text-based therapy would be best implemented as a way to supplement, rather than replace, face-to-face services.

**Conclusions:**

Altogether, the results of this study suggest that text-based therapy holds the potential to increase access to and use of mental and behavioral health services; however, graduate students remain concerned about its effectiveness and the optimal methods of implementation. Future research should investigate how therapeutic processes (eg, effective communication and rapport-building) can be facilitated in digital environments and how text-based therapy could be best implemented to supplement and extend, rather than replace, face-to-face services.

## Introduction

Approximately 18.9% of all adults in the United States live with a mental illness, with the highest prevalence rates observed among young adults aged 18 to 25 years [1,2]. Mental health disorders account for approximately one half of the disease burden among young adults [3] and, when compared with neurological and substance use disorders, constitute the highest proportion of disability-adjusted life years [4]. Graduate students represent a particularly vulnerable population, with data indicating greater risk of depression and anxiety compared with the general population [5,6]. Therefore, many researchers have argued that there is a mental health crisis occurring in this population [5].

In-person therapy remains the most widely used method to treat individuals with mental and behavioral health conditions [7]. Across several one-on-one or group sessions, trained therapists aim to help patients understand their thoughts, feelings, and patterns of behavior while identifying ways to positively cope with psychosocial stressors [8,9]. Therapy has been proven to be effective in treating common mental and behavioral health disorders, with substantial evidence supporting its effectiveness for decreasing symptoms of major depressive disorder, generalized anxiety disorder, and panic disorder in adult populations [7,10].

There are several barriers experienced by undergraduate and graduate students related to the use of mental and behavioral health services [11,12]. Individual-level barriers include scheduling conflicts, lack of time, negative self-stigma associated with seeking therapy, financial constraints, lack of perceived need, and perceived ineffectiveness of treatment [13]. Organizational-level barriers include a lack of insurance coverage for mental and behavioral health services and extended waitlists for appointments on campus [11]. Innovative delivery formats, such as delivery of treatment through the internet or mobile devices, have the potential to address several of these barriers and thus increase graduate students’ use of mental health services. These approaches may be particularly well suited for this population as technology has a strong presence among young adults and students on university campuses [11,14].

The delivery of therapy via SMS text message, in particular, has been gaining momentum [15]. Text-based therapy relies on asynchronous communication and allows individuals to send written messages to a certified counselor or a therapist using a mobile device. The therapist then responds (via the same platform) at their earliest convenience [15]. Despite its increasing popularity [16], research on the efficacy and acceptability of text-based therapy has been limited. One study demonstrated that adding SMS text messages to ongoing in-person therapy did not improve clinical outcomes in adult populations [17]; however, another found that the use of a text-based therapy service for 15 weeks produced significant reductions in symptoms and increased work productivity in adults with depression and anxiety [18]. In terms of acceptability, 1 study found that asynchronous text therapy with a licensed therapist was viewed as acceptable and clinically beneficial for adults with various diagnoses and histories of psychological distress [19]. To date, no studies have investigated the acceptability of text-based therapy in graduate students.

To address this gap, this study aimed to survey current graduate students at a large southeastern university in the United States regarding their awareness, acceptability, and perceived effectiveness of text-based therapy for treating mental and behavioral health challenges. Moreover, this survey aimed to identify which factors would promote or prevent the use of text-based therapy within this specific population. Finally, this study examined whether there were differences in the likelihood of seeking out face-to face and text-based therapy by age, race, gender, and sexual orientation. On the basis of the results from a previous study, which found that White individuals were more likely than Hispanic, Black, or African American individuals to receive mental health treatment [20], it was hypothesized that individuals who were younger, female, heterosexual, and non-Hispanic White would be more likely to seek out therapy than their respective counterparts.

## Methods

### Recruitment

This study aimed to recruit current graduate students at both the master and doctoral level who were employed by their departments at the University of Florida in exchange for a stipend (considered *graduate assistants* at the University of Florida). Potential participants were eligible to complete the survey if they were a current graduate assistant, were aged ≥18 years, and were enrolled in GatorGradCare, an employer-sponsored health plan specific to graduate assistants at the University of Florida. At the time that the current survey was conducted (spring 2019), GatorGradCare had started offering free asynchronous text-based therapy (via TalkSpace) as part of their health insurance plan benefits.

To recruit participants for this study, the first author (SAB, who at the time was completing a public health internship at GatorCare) sent an email on January 21, 2019, to all current University of Florida graduate assistants through the GatorGradCare listserv. This email introduced the purpose of the study and provided participants with a link to complete the study survey through Qualtrics, a web-based survey platform. Participants were asked to read a statement that outlined the purpose of the project; instructions describing how to complete the survey; and information about potential risks and benefits, confidentiality, voluntary participation, and participant rights (eg, the right to refuse to answer individual questions and to withdraw participation at any time). Individuals who provided web-based consent to participate were prompted to complete the remainder of the survey questions. On February 1, 2019, 2 weeks after the initial email was sent, the first author sent a second follow-up email to the GatorGradCare listserv, to serve as a reminder for those who may have still wanted to participate. The survey closed 1 month following initial contact (February 15, 2019). No compensation was provided for completing the survey.

### Measures

The survey used in this project consisted of 19 self-report items (with a mix of multiple-choice and open-ended questions) developed by the first author (SAB). Questions are described by content area in subsequent sections.

### Demographics

Participants were asked to report their age, race, gender, and sexual orientation. No personal identifiers (eg, name, address, or email address) were collected from the survey.

### Experiences With Traditional Face-to-Face Therapy

Participants were asked if they had previously used mental and behavioral health services (defined as “therapy or counseling”) and, if so, for what concerns. Participants could check all that applied from the following options: substance abuse, alcohol abuse, anxiety, depression, stress management, attention-deficit/hyperactivity disorder, gender dysphoria, relationships, eating disorders, sexual assault, smoking cessation, autism spectrum disorder, or bereavement (these terms were used as they were listed as example conditions for treatment by the TalkSpace text-based therapy service [21]). There were also options of “prefer not to answer” and “other,” which included a text box that enabled participants to elaborate further. Participants were also asked about barriers that prevented them from using therapy or counseling services in the past, perceived effectiveness of face-to-face therapy, and their likelihood of seeking out face-to-face therapy. Perceived effectiveness of face-to-face therapy was assessed using a 5-point Likert scale ranging from 1=*extremely effective* to 5=*not effective at all*. The likelihood of seeking out face-to-face therapy was also assessed on a 5-point Likert scale ranging from 1=*very likely* to 5=*not likely at all*.

### Experiences With Text-Based Therapy

Participants were asked if they were aware of text-based therapy before the onset of the study, if they would consider using text-based therapy if available, the factors that would promote them to use text-based therapy, the factors that would promote them to not use text-based therapy, their likelihood of seeking out text-based therapy, and perceived effectiveness of text-based therapy. Perceived effectiveness of text-based therapy was assessed using a 5-point Likert scale ranging from 1=*extremely effective* to 5=*not effective at all*. The likelihood of seeking out text-based therapy for behavioral health issues was also assessed on a 5-point Likert scale ranging from 1=*very likely* to 5=*not likely at all*.

### Open-Ended Survey Questions

Participants were asked to provide free-text responses to three open-ended questions: (1) “Do you think text-based counseling can be effective? Why or why not? What factors do you think limit its effectiveness?” (2) “Do you see any advantages to using text-based counseling over face-to-face counseling? Disadvantages?” (3) “Please provide any additional comments regarding your perception of text-based therapy.”

### Data Analysis

All quantitative analyses were conducted using SPSS Statistics (version 25; IBM Corp). Descriptive statistics were used to summarize the demographic and quantitative data. Chi-square tests and ANOVAs were used to examine whether there was an association between past use of therapy or counseling and willingness to use text-based therapy in the future, and if this association varied by age, race, gender or sex, or sexual orientation. Nonparametric Wilcoxon signed-rank tests were conducted to assess whether there were significant differences between the perceived effectiveness and likelihood of seeking therapy between text-based and face-to-face therapy services.

The constant comparative method, which is used by researchers to uncover similarities, differences, and patterns in data [22], was used for qualitative analyses of the responses from the 3 open-ended questions. Open coding methods [22] were used to examine, compare and contrast, and categorize the raw data. Data were then compiled into subthemes, and these subthemes were then compiled into larger, overarching themes. Coding was done individually by two authors (SAB and ANB) to limit subjectivity and bias. SAB and ANB then compared results of this coding process, with disagreements resolved through a consensus discussion and review by KMR.

### Ethics Approval

This project was approved by the University of Florida Institutional Review Board (IRB#201802501).

## Results

### Participants

Of the 3609 graduate assistants who were contacted via the GatorGradCare listserv, 297 provided consent to participate in this study (reflecting a response rate of 8.2%). Responses from 32 participants were excluded owing to extensive missing data (ie, completing less than half of the survey questions), resulting in 265 participants being included in the current analyses. Demographic characteristics of survey participants are provided in Table 1. On average, participants were aged 28.3 (SD 5.1) years, and the majority identified as non-Hispanic White (166/265, 62.6%), female (167/265, 63%), and heterosexual or straight (215/265, 81.1%).

**Table 1:** Participant demographics (N=265).

Characteristic		Value
Age (years; n=245), mean (SD)		28.3 (5.1)
**Race and ethnicity, n (%)**		
	Asian, non-Hispanic	32 (12.1)
	Black or African American, non-Hispanic	12 (4.5)
	Hispanic or Latinx	28 (10.6)
	White, non-Hispanic	166 (62.6)
	Another race or multiple races selected	22 (8.3)
	Prefer not to answer or missing	5 (1.9)
**Gender, n (%)**		
	Female	167 (63)
	Male	87 (32.8)
	Transgender	3 (1.1)
	Other	1 (0.4)
	Prefer not to answer or missing	7 (2.6)
**Sexual orientation, n (%)**		
	Heterosexual or straight	215 (81.1)
	Bisexual	20 (7.6)
	Homosexual	12 (4.5)
	Another category	9 (3.4)
	Prefer not to answer or missing	9 (3.4)

### Past Utilization of Behavioral Health Services and Awareness of Text-Based Therapy

Approximately 60.4% (160/265) of survey participants reported previous use of some form of mental or behavioral health services. The top three concerns individuals sought face-to-face therapy for were anxiety, depression, and stress management (Table 2). Additional concerns that participants mentioned previously seeking face-to-face therapy for included eating disorders, bereavement, parental divorce, posttraumatic stress disorder, bipolar disorder, and suicidal ideation. Individuals who identified with historically marginalized racial or ethnic groups were significantly less likely to report having used mental or behavioral health services compared with non-Hispanic White participants (45/94, 48%, vs 112/166, 67.5%, respectively; n=260, χ^2^_1_=9.6; *P*=.002). Moreover, female students were more likely to report having used these services in the past than male students (113/167, 67.7%, vs 38/87, 44%, respectively; n=254, χ^2^_1_=13.7; *P*<.001). There were no significant differences in reports of past use by sexual orientation (n=256, χ^2^_2_=2.3; *P*=.34) or age (*F*_1,245_=2.6; *R*^2^=.01; *P*=.10).

**Table 2:** Top 5 concerns for which individuals sought face-to-face therapy.

Condition	Participants who sought out face-to-face therapy for this condition, n (%)
Anxiety	110 (41.5)
Depression	105 (39.6)
Stress management	70 (26.4)
Relationships	45 (16.9)
Sexual assault	20 (7.5)

Approximately half of participants (139/265, 52.5%) were not aware that text-based counseling existed before participating in this study; however, 65.3% (173/265) of participants reported they would consider using text-based counseling if available. Participants reported they would most likely seek text-based therapy for concerns such as stress management, anxiety, and depression (Table 3).

**Table 3:** Top 5 concerns for which individuals would most likely seek text-based therapy.

Condition	Participants who reported that they would likely seek text-based therapy for this condition, n (%)
Stress management	136 (51.3)
Anxiety	131 (49.4)
Depression	110 (41.5)
Relationships	81 (30.5)
Sexual assault	23 (8.6)

### Barriers to Accessing Face-to-Face and Text-Based Therapy Services

The top barriers that prevented participants from seeking mental and behavioral health services included cost (115/265, 43.4%), scheduling conflicts (107/265, 40.4%), that it was not needed (70/265, 26.4%), and that a therapist could not be found (66/265, 24.9%). In total, 50/265 (18.9%) participants also reported “other” barriers and wrote responses including anxiety about starting therapy, stigma, long wait times owing to an insufficient number of counselors, and insufficient coverage by insurance.

The top factors that would promote participants to seek text-based therapy were convenience (206/265, 77.7%), low cost (141/265, 53.2%), anonymity from other individuals in person (76/265, 28.7%), and a better sense of privacy and security (70/265, 26.4%). In total, 29/265 (10.9%) participants described “other” reasons promoting the use of text-based therapy, including preferences for communicating via text versus in person and anxiety regarding face-to-face communication. Barriers that would prevent participants from seeking text-based therapy were a preference for face-to-face contact (189/265, 71.3%), that they were not interested or did not need therapy (58/265, 21.9%), cost (40/265, 15.1%), and a preference for anonymity related to privacy and security (20/265, 7.5%). In total, 42/265 (15.8%) “other” barriers were reported, including the impersonal nature of text-based communications, perceptions that text-based communication would prevent development of a deep relationship between therapist and client, and a perceived lack of effectiveness.

### Perceived Effectiveness and Likelihood of Seeking Face-to-Face Versus Text-Based Psychotherapy Services

**Figure 1:**
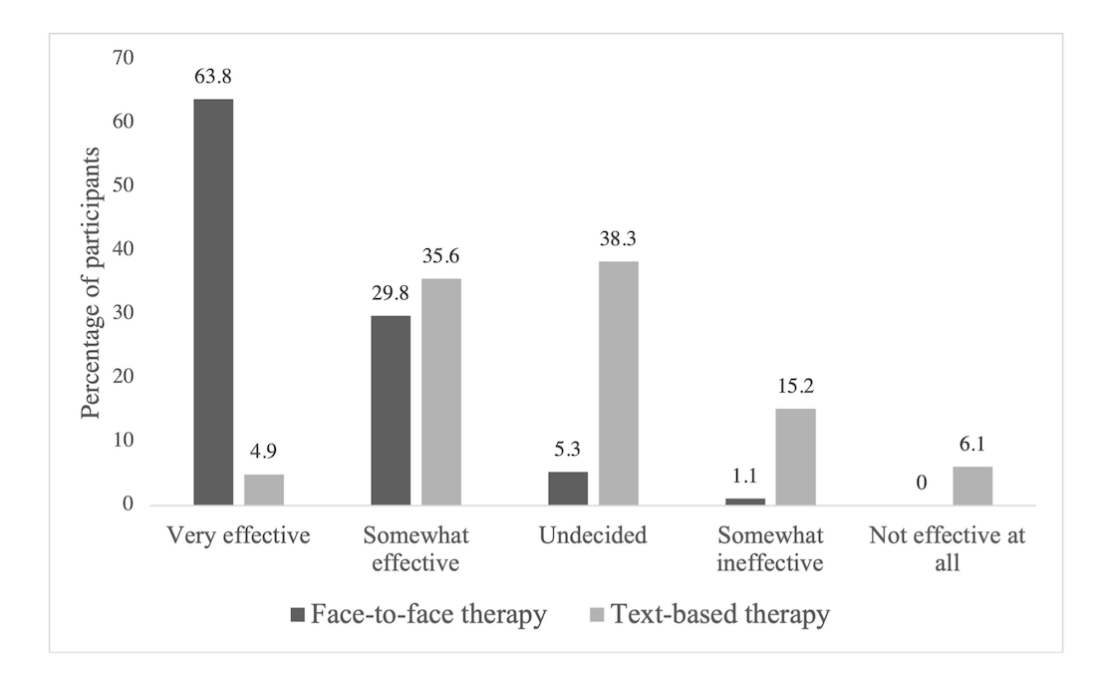
Participants’ perceived effectiveness of face-to-face versus text-based therapy.

**Figure 2:**
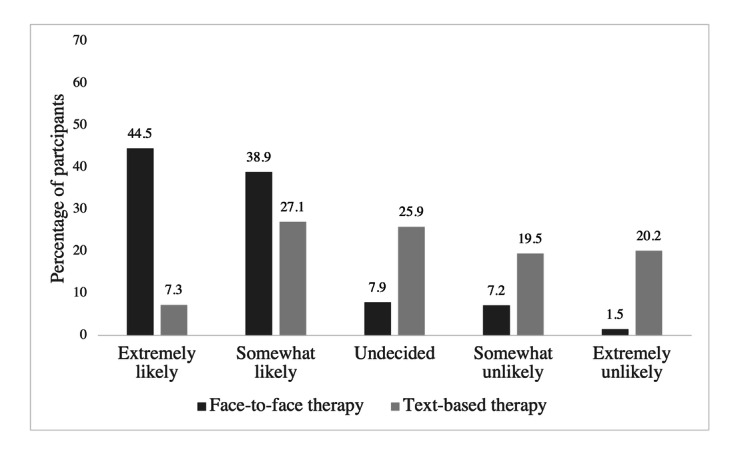
Participants' reported likelihood of seeking face-to-face versus text-based therapy.

Figure 1 provides ratings of perceived effectiveness of therapy by modality. Most participants (169/265, 63.8%) reported beliefs that face-to-face therapy was "very effective" in treating mental and behavioral health concerns. In comparison, only 13/265 (4.9%) participants reported that text-based therapy was “very effective”; the remaining participants reported beliefs that text-based therapy was “somewhat effective” or that they remained “undecided.” Figure 2 provides responses related to the likelihood of seeking therapy by each delivery modality. Although most participants (221/265, 83.4%) reported that they were “extremely likely” or “somewhat likely” to seek face-to-face therapy, only 34.4% (90/262) of participants responded similarly for text-based therapy. Participants rated the perceived effectiveness of face-to-face therapy significantly higher than text-based therapy (*z*=−12.33; n=264; *P*<.001) and reported a significantly higher likelihood of seeking face-to-face therapy than text-based therapy (*z*=10.69; n=262; *P*<.001). There was no significant association between past use of behavioral health services and willingness to use text-based therapy (N=265, χ^2^_1_=0.0; *P*=.91). Moreover, no significant differences in likelihood of seeking text-based therapy were found by age (*F*_1,243_=1.70; *R*^2^=.01; *P*=.19), race (n=260, χ^2^_4_=6.0; *P*=.20), sexual orientation (n=247, χ^2^_2_=1.2; *P*=.54), or gender (n=257, χ^2^_2_=0.0; *P*=.99).

### Qualitative Results

Participants provided 533 responses across the 3 open-ended survey questions, from which 1090 data points were coded, reduced to 36 subthemes, and then 6 broader overarching themes (Table 4). The 6 themes that emerged are described in the following sections.

**Table 4:** Themes and subthemes developed from open-ended questions assessing participants’ perceptions of text-based therapy.

Theme		Mentions, n (%)
**Factors that promote the use of text-based therapy (advantages)**		398^a^ (36.51)
	Convenience	215 (19.72)
	Provides anonymity	42 (3.85)
	Less costly	39 (3.57)
	Mitigates social anxiety	37 (3.39)
	Technological features	23 (2.11)
	Easier to express thoughts	18 (1.65)
	Time efficient	16 (1.46)
	Less stigma than face-to-face therapy	4 (0.36)
	Past positive experiences with text-based therapy	4 (0.36)
**Factors that dissuade the use of text-based therapy (disadvantages)**		325^a^ (29.81)
	Does not provide robust therapy experience for patients	159 (14.58)
	Stunted communication	141 (12.93)
	Privacy and security risks	9 (0.82)
	Unsure of how it works	6 (0.55)
	Technological barriers	5 (0.45)
	Past negative experiences with text-based therapy	5 (0.45)
**Perceived effectiveness of text-based therapy**		210 (19.26)
	Potential to be effective	61 (5.59)
	Face-to-face therapy is more effective	43 (3.94)
	Not an effective mode of communication	37 (3.39)
	Effective in some scenarios	30 (2.75)
	Unsure about effectiveness	23 (2.11)
	Effective for younger populations	11 (1.00)
	More effective than no counseling	5 (0.45)
**Overall perception of text-based therapy**		94 (8.62)
	Increases access to mental health services	32 (2.93)
	Avoids providing adequate and effective mental health care	24 (2.2)
	Support implementation	23 (2.11)
	Potential to be helpful	10 (0.91)
	Oppose implementation	5 (0.45)
**Recommendations to improve effectiveness of text-based therapy**		34 (3.11)
	Use as a supplement to face-to-face therapy	21 (1.92)
	Various options to communicate electronically with therapist	8 (0.73)
	Have consistent counselors	2 (0.18)
	Use as a way to triage patients	2 (0.18)
	Need to establish clear boundaries	1 (0.09)
**Likelihood of using text-based therapy**		29 (2.66)
	Would not use it	19 (1.74)
	Would use it	7 (0.64)
	Would want a test trial before committing	2 (0.18)
	Would use as a last resort	1 (0.09)

^a^Each participant could answer more than once; thus, the total may exceed 265.

### Factors That Promote the Use of Text-Based Therapy

The most common overarching theme focused on factors that promote the use of text-based therapy (398/1090, 36.51%), which had 9 subthemes. Ease of finding a therapist, ability to use text-based therapy anywhere, and avoidance of long wait times at the counseling center or other providers’ offices were all elements mentioned by participants when referring to the convenience of text-based therapy. For example, 1 participant explained:

Text-based is far more immediate. If you’re struggling with something, you can get a response much more quickly. There’s also a lower activation energy to setting up that service than to getting an appointment with a counselor face-to-face.

Another said, “It would be much easier to schedule around daily life activities (as a grad student, I don’t have much free time).”

### Factors That Dissuade the Use of Text-Based Therapy

The second most common theme represented factors that would dissuade participants from using text-based therapy (325/1090, 29.81%), which included 6 subthemes. Responses noted that face-to-face therapy offers a “robust” experience including a personal setting, engagement from both parties, and a strong therapist-client relationship. In contrast, it was mentioned that these important characteristics are lost within text-based therapy, with one participant saying:

I believe that the key to the counselor/patient relationship is face-to-face empathy and rapport building. This might be more difficult to achieve via text.

The lack of nonverbal communication was also frequently mentioned as a barrier to text-based approaches, as both therapists and patients would be unable to express and read nonverbal cues including facial expressions, emotions, and body language, leading to misinterpretation of text messages and stunted communication. This sentiment was reflected in the following participant response:

Text messaging simply does not convey all the same cues, signals, and emotions as face-to-face communication (e.g., voice inflexion; body language). It is not as effective a mode of communication. I am not convinced that a therapist would be able to make an accurate diagnosis or to have as deep of insights over text as they would if they were able to observe their patients face-to-face. I also do not think that I would be able to express myself and my feelings as well via text as I would in person, even if I tried.

### Perceived Effectiveness of Text-Based Therapy

The third most common theme represented participants’ perceptions regarding the effectiveness of text-based therapy (210/1090, 19.26%) and included 7 subthemes. The effectiveness of text-based therapy was described as being dependent on the complexity of the issue and personal preferences of the individual seeking care. For example, 1 participant said, “For basic, straightforward issues such as stress management techniques it might be fine, but for more complex issues I think the connection of direct face-to-face communication would be better and more effective.”

Similarly, another participant stated:

When it comes down to mental health issues, I would like to speak to someone in person. So, in this case, technology get in the way in this case.

### Overall Perception of Text-Based Therapy

The fourth most common theme focused on how participants perceived the implementation of text-based therapy as a new and potentially viable option (94/1090, 8.62%). Some participants perceived text-based therapy as a step in the right direction for increasing access to and reach of services, filling a gap in areas where there is a lack of available counselors. One participant said:

For students who do not have access to counseling in person, it will be very advantageous and for students who do not like to meet in person. I see its advantages and support the effort on campus.

Conversely, many participants viewed text-based therapy as a cost-cutting measure that the university and other organizations may be using to avoid the problem of not having enough counselors available for use:

I appreciate that this can broaden the scope of services for graduate students and other populations (e.g., rural populations) but I also have concerns it can be a band-aid that allows people to disregard the larger problem of major disparities in access to adequate healthcare.

### Recommendations to Improve Effectiveness of Text-Based Therapy

The fifth most common theme included various strategies participants recommended to improve the effectiveness of text-based therapy (34/1090, 3.1%). Participants stated that text-based therapy should be used to augment, and not replace, face-to-face therapy. For example, 1 participant stated:

I think it has potential to help but that’s not to say that we should be trying to do away with face-to-face counseling. This should just be something supplemental. Like if therapists/psychologists have “on-call hours” when their clients can text.

Another stated:

Something is better than nothing. Not a big fan of the idea, but if that is the way to facilitate mental health support throughout the system, it is a good idea to implement it side by side with the traditional face-to-face resources.

In addition, some participants reported they would like to see text-based therapy platforms include different features to communicate electronically, including video or audio chat with a therapist and group-based counseling.

### Likelihood of Using Text-Based Therapy

The least common theme that emerged illustrated participants’ likelihood of using text-based therapy (29/1090, 2.7%), which had 4 subthemes. When asked why they would not use text-based therapy, some participants referenced that they “hated texting” and others noted that they would not want to use their phone when experiencing behavioral health issues. One participant said, “I would never use text-based counseling because I don’t feel comfortable putting private, sensitive information in writing.”

Conversely, other participants noted that they would use text-based therapy because they used technology for everything else in their lives, with 1 participant stating:

I think it is a great idea especially as younger generations are becoming more and more dependent on technology and prefer communicating via text message. In addition, it allows the opportunity for individuals with very busy schedules to be able to get help.

## Discussion

### Principal Findings

The aim of this study was to assess awareness and acceptability of text-based therapy for the treatment of mental and behavioral health concerns in graduate students. Approximately half of the participants reported not being aware that text-based therapy services existed, despite existing coverage by their insurance plan. Promisingly, two-thirds of participants said they would consider trying text-based therapy. Qualitative results demonstrated that participants found text-based therapy services convenient and less costly than traditional face-to-face services and that participants thought that these services provided beneficial anonymity.

Despite most participants reporting that they would consider using text-based therapy, participants were significantly less likely to report that they would seek these services compared with face-to-face therapy. This may have been driven by differences in perceived effectiveness, as participants perceived text-based therapy as significantly less effective than face-to-face therapy. Qualitative results revealed that participants had concerns over the ability of patients and therapists to develop rapport given the inability to express emotion and mood through nonverbal cues (eg, eye contact, body posture, and tone of voice). Given that rapport, or therapeutic alliance, has been established as one of the most influential factors in the success of therapy [23], this could be a key driver of the lower rates of perceived effectiveness of text-based therapy. However, there is emerging evidence that rapport can be successfully developed through text-based therapy [24] because of factors unique to text-based communication. For example, the web-based disinhibition effect may allow individuals to feel safe sharing things through digital means that they would not otherwise feel comfortable sharing in person [25,26]. Interestingly, participants in web-based therapy have reported both similar [27] and significantly higher [28] levels of therapeutic alliance than levels reported by participants in face-to-face therapy. Future work should continue to examine how the digital environment can be used to better facilitate therapeutic processes.

Qualitative results also indicated that the type of mental and behavioral health concern may also affect likelihood to engage with text-based therapy services, as text-based therapy was perceived by some respondents as more effective for some concerns versus others. Nevertheless, participants reported similar concerns for which they would seek face-to-face and text-based therapy services. Overall, the top three concerns for which participants said they would seek text-based therapy were stress management, depression, and anxiety. Given that these conditions are the most common mental health concerns in graduate students [5,29] and research demonstrating that text-based therapy can reduce anxiety and depression levels in young adults [30,31], the current results suggest that text-based therapy has potential to increase access to and the use of mental and behavioral health services among graduate students.

Approximately 60% of respondents reported previous use of behavioral health services before the onset of this study, with a significantly higher proportion of female (vs male) and non-Hispanic White (vs racial or ethnic minority) participants reporting previous use of behavioral health services. These results were consistent with lower rates of health care use observed among men and individuals from racial or ethnic minority groups in previous studies [32-34]. In contrast, there were no differences in likelihood of seeking text-based therapy by age, race or ethnicity, sexual orientation, or gender (and no association was found between past use of mental or behavioral health services and likelihood of using text-based therapy), suggesting this modality may also increase access to and use of mental and behavioral health services by individuals in groups that have historically demonstrated lower rates of use of face-to-face services [35,36].

Overall, qualitative results demonstrated that participants perceived text-based therapy as having potential to increase access to mental and behavioral health services; however, caution was suggested at the use of text-based therapy to replace, rather than supplement, face-to-face services. Participants noted that text-based therapy would be best implemented as a method of extending access to services (eg, providing services to individuals who experience barriers to attending traditional face-to-face sessions), but that face-to-face services should still be offered, especially for students experiencing more complex mental or behavioral health challenges. Some concern was also expressed regarding the use of text-based therapy as a “Band-Aid” in place of larger systemic changes that may be necessary to address existing disparities in access to mental and behavioral health services.

### Strengths and Limitations

There were several strengths of this study. First, this study was the first to assess perceptions of text-based therapy services in graduate students, a key population at high risk for the development (and worsening) of mental and behavioral health conditions [5]. Second, the mixed methods approach provided a deeper understanding of participants’ perceptions of text-based therapy than would have been possible using only quantitative analysis approaches, allowing us to not only observe differences in perceived effectiveness between the services but also to provide potential avenues for future research and implementation. Finally, data were coded independently by two researchers (SAB and ANB), which minimized the impact of individual bias on thematic coding.

This study also had important limitations. The sample was comprised primarily of heterosexual, non-Hispanic White women, which limits the generalizability of results. The University of Florida has a status as a predominately White institution, with 56.6% of students identifying as White in 2019 [37]. Thus, the results may not be generalizable to institutions of other classifications, such as historically Black colleges or universities. In addition, the sample included only graduate assistants at the University of Florida. These graduate students have a position that funds their education costs and provides a health insurance plan. It is possible that there are differences in perceptions of text-based counseling between graduate assistants who have the GatorGradCare insurance plan versus other graduate students who do not work for the university, who have other forms of insurance, or who have no health insurance at all. It is also unclear how these perceptions may differ by socioeconomic status, which is a variable worth exploring in future studies. Finally, the low rates of awareness of text-based therapy may be attributed to the novelty of the modality and the recent availability of the service; text-based therapy services became available within GatorCare only 3 months before the initiation of the study [38]. GatorCare used a variety of different methodologies to increase awareness surrounding this benefit, including tabling at the new graduate student orientation and sending an email campaign to GatorGradCare subscribers; however, it appears that these initiatives were only partially successful in increasing awareness of the benefit and of text-based therapy as a therapy modality.

### Conclusions

This study provided novel insights regarding perceptions of text-based therapy among graduate students. Most participants reported that they would consider using text-based therapy; however, participants reported that they were less likely to seek these services compared with traditional face-to-face therapy. Although participants felt that text-based therapy had several advantages (eg, the ability to increase access to mental and behavioral health services in both a convenient and cost-efficient way), most perceived text-based therapy as less effective than traditional face-to-face therapy. Altogether, results suggest that text-based therapy may hold potential to increase access to and use of mental and behavioral health services but that concerns remain regarding its effectiveness and how it should best be implemented. Future research should focus on methods to facilitate effective therapeutic processes in digital environments (eg, using aspects of communication unique to digital mediums to improve communication of emotion, mood, and tone) and on how text-based therapy services could be best implemented to supplement and extend, rather than replace, face-to-face services.
